# Serum Biomarkers for the Prediction of Hepatocellular Carcinoma

**DOI:** 10.3390/cancers13071681

**Published:** 2021-04-02

**Authors:** José D. Debes, Pablo A. Romagnoli, Jhon Prieto, Marco Arrese, Angelo Z. Mattos, André Boonstra, on behalf of the ESCALON Consortium

**Affiliations:** 1 Department of Gastroenterology and Hepatology, Erasmus MC Rotterdam, 3015 CE Rotterdam, The Netherlands; 2 Department of Medicine, University of Minnesota, Minneapolis, MN 55455, USA; 3 Centro de Investigaciones en Medicina Translacional “Severo Amuchastegui” (CIMETSA), Instituto Universitario de Ciencias Biomédicas de Córdoba (IUCBC), Córdoba 5016, Argentina; pablo.romagnoli@iucbc.edu.ar; 4 Centro de Enfermedades Hepaticas y Digestivas, Bogota CS412, Colombia; jhonprieto@hotmail.com; 5 Department of Gastroenterology, Escuela de Medicina, & Centro de Envejecimiento y Regeneración (CARE), Pontificia Universidad Católica de Chile, Santiago 8330077, Chile; marrese@med.puc.cl; 6 Graduate Program in Medicine: Hepatology, Universidade Federal de Ciências da Saúde de Porto Alegre, Porte Alegre 90050-170, Brazil; angeloz@ufcspa.edu.br

**Keywords:** hepatocellular carcinoma, biomarker, cytokines, microRNA, tumor marker

## Abstract

Hepatocellular carcinoma (HCC) is a leading cause of global cancer death. Major etiologies of HCC relate to chronic viral infections as well as metabolic conditions. The survival rate of people with HCC is very low and has been attributed to late diagnosis with limited treatment options. Combining ultrasound and the biomarker alpha-fetoprotein (AFP) is currently one of the most widely used screening combinations for HCC. However, the clinical utility of AFP is controversial, and the frequency and operator-dependence of ultrasound lead to a variable degree of sensitivity and specificity across the globe. In this review, we summarize recent developments in the search for non-invasive serum biomarkers for early detection of HCC to improve prognosis and outcome for patients. We focus on tumor-associated protein markers, immune mediators (cytokines and chemokines), and micro-RNAs in serum or circulating extracellular vesicles and examine their potential for clinical application.

## 1. Introduction

Hepatocellular carcinoma (HCC) is the most common primary liver malignancy. According to current epidemiological data, it is the fourth leading cause of cancer mortality worldwide, ranking among the most commonly diagnosed cancer in both genders [1,2]. HCC generally develops in the context of liver cirrhosis of any cause. However, it is particularly linked to infections with the hepatitis B virus (HBV) or hepatitis C virus (HCV) and alcoholic or nonalcoholic fatty liver disease (NAFLD), which are the most common underlying etiologies [2,3,4].

**Figure 1:**
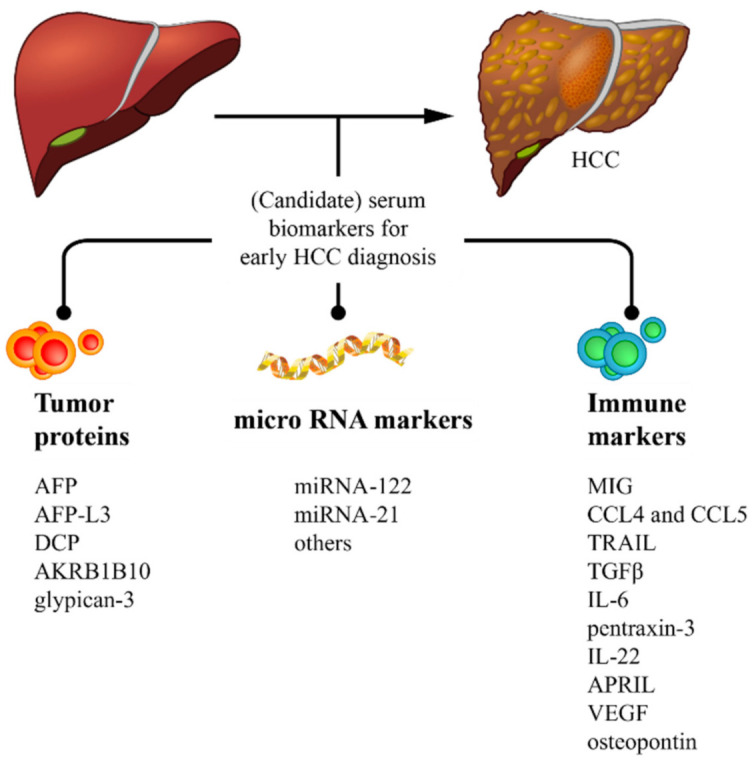
Graph summarizing different classifications of biomarkers discussed in the review. For abbreviations, please see the main text

HCC-related mortality has steadily increased and almost tripled in the United States since the 1980s, where it is the fastest rising cause of cancer-related deaths with more than 39,000 cases and 29,000 deaths in 2018 [5]. Importantly, according to World Health Organization estimates, globally, more than one million patients will die from liver cancer in 2030 [6]. These data underscore the magnitude of the HCC-associated disease burden, which, despite the advances made in its surveillance and diagnosis, is still often diagnosed at advanced stages, precluding timely and eventually curative therapeutic intervention resulting in poor prognosis. Thus, early HCC diagnosis is critical in order to improve patient outcomes. Proposed strategies for early detection of HCC include adherence to surveillance programs in populations at risk and the development of sensitive and specific diagnostic biomarkers [7,8]. HCC surveillance comprising of ultrasound screening every six months is recommended for all patients with cirrhosis, but, as mentioned above, tailoring HCC surveillance programs may be necessary for certain diseases (i.e., HBV and NAFLD) so to include at-risk non-cirrhotic patients [9]. In this regard, non-cirrhotic HBV-infected individuals are advised to undergo surveillance in a range of ages based on geographic location across the globe. Participation in surveillance for HCC in cirrhotic individuals has been regarded as suboptimal, with some studies in the United States and Europe showing that less than 40% of patients with cirrhosis undergo proper HCC surveillance [10,11]. A large study from our group involving over 1300 HCC cases in South America found that less than 50% of HCCs were diagnosed via surveillance [12]. Although several issues contribute to such low participation in surveillance, the lack of a reliable blood biomarker that is easy to detect and that provides a high degree of sensitivity and specificity is certainly among the most important contributing factors. Due to the diverse genetic nature of HCC and the existence of different underlying liver diseases, no biomarker has progressed to the point of implementation. Underlying liver disease is indeed a point of contention as in processes like viral hepatitis; there is an ongoing inflammatory cascade that obscures the approach to identifying immune or inflammatory markers that could be related to the tumor. With new advancing technologies, which allow us to detect low expressing proteins, RNAs and genetic material in endovesicles, a number of new circulating biomarkers are currently under study [8,13]. In this review, we aim to summarize the available information and recent developments on HCC biomarkers detected in serum, classified as protein, miRNA and immune biomarkers (Figure 1), which may impact early HCC diagnosis and, therefore, implementation of appropriate management that can optimize prognosis in HCC. We aim to present what are considered to be the most advanced, important or novel biomarkers and not to present an exhaustive review.

## 2. Protein Biomarkers

Alpha-fetoprotein (AFP) is the most commonly used biomarker for the surveillance and diagnosis of HCC. Nevertheless, current guidelines either do not recommend its use or make it optional in addition to ultrasound [14,15]. The main reasons that prevent the widespread use of AFP are concerning the specificity and limited sensitivity it has in detecting early-stage HCC, with a considerable number of HCC that do not have elevated serum AFP levels. Elevated AFP serum levels are also observed in some patients with viral hepatitis, cholangiocarcinoma and other tumors, leading to false-positive results for HCC diagnosis [16]. However, a meta-analysis on HCC surveillance has demonstrated that ultrasound alone is less sensitive than ultrasound associated with AFP (sensitivity of 45% versus 63%, relative risk of 0.81 for early-stage HCC in patients with cirrhosis) [17]. Also, a model based on the pattern of increase of AFP over time identified patients at high risk of developing HCC as early as 15 years before the diagnosis so that these individuals could be monitored more intensively than their counterparts. The area under the receiver operating characteristic curve (AUROC) for AFP ranged from 0.73 to 0.83 in different cohorts [18].

As the performance of AFP is suboptimal, many other serum biomarkers are under investigation. Two markers that received much attention are a glycoform of AFP, lectin-binding AFP-3 (AFP-L3), and des-gamma-carboxyprothrombin (DCP), also known as prothrombin induced by vitamin K absence-II (PIVKA-II), which is a non-functional protein produced by HCC. A randomized controlled trial evaluating HCC surveillance through ultrasound with or without AFP, AFP-L3 and DCP has demonstrated that the association of these biomarkers with ultrasound increases sensitivity while decreasing specificity [19]. In another study in which samples from four prospective Korean cohorts were analyzed, AFP performed better than AFP-L3 and DCP up to 12 months prior to the diagnosis of HCC. When biomarkers were combined, the association between AFP and AFP-L3 had the best performance both at 12 and at 6 months before the diagnosis. At the moment of the diagnosis, AFP combined with AFP-L3 performed comparably as the combination of all three biomarkers. Regarding early-stage HCC, results were also favorable to AFP combined with AFP-L3. Finally, adding AFP and AFP-L3 to ultrasound improved the sensitivity (94.3%), despite decreasing specificity (82.7%) [20].

A score, which includes these biomarkers is the GALAD score, an acronym for gender, age, AFP-L3, AFP and DCP, which resulted in AUROC values of more than 0.88 irrespective of the HCC disease stage [21]. In a North American validation cohort, the score had an AUROC of 0.95 for HCC detection, while ultrasound had an AUROC of 0.82, a superiority that remained for early-stage as well as very early-stage HCC. In the same study, the GALADUS score was described, adding ultrasound findings to the GALAD score. The GALADUS score had AUROCs of 0.98 for any-stage HCC and 0.97 for early-stage HCC [22]. In patients with nonalcoholic steatohepatitis, the GALAD score demonstrated sensitivity and specificity with an AUROC of 0.96 to identify patients with any stage HCC Importantly, these high AUROC values were observed in NASH patients with and without cirrhosis (0.93 and 0.98, respectively). The GALAD score also identified individuals who would develop HCC as early as 1.5 years before the diagnosis. When early-stage HCC was concerned, the score had a sensitivity of 86.2% and a specificity of 90.9% [23].

In addition, different classes of serum protein biomarkers have been described. The LCR1 model identifies patients without cirrhosis who are at risk of developing primary liver cancer. The parameters that makeup LCR1 include serum apolipoprotein A1, haptoglobin, gamma-glutamyltranspeptidase, alpha2-macroglobulin, age and gender. In addition, a second model has been described, the LCR2 model, which is used to follow individuals identified by LCR1 and patients with cirrhosis; LCR2 includes the same variables combined with AFP. AUROCs were 0.78 for LCR1 and 0.87 for LCR2, and LCR2 performed better than AFP (AUROC = 0.72) [24].

Another panel of serum biomarkers for early diagnosis of HBV-related HCC consists of five plasma proteins (P5). The P5 panel includes osteopontin, growth and differentiation factor 15 (GDF15), neuron-specific enolase, thrombin receptor activator for peptide 5 and osteoprotegerin. The panel outperformed AFP in the diagnosis of early-stage HCC (AUROC = 0.85–0.91 for P5 and 0.54–0.59 for AFP, according to different cutoff values and different validation cohorts). Furthermore, P5 predicted HCC development approximately one year before being clinically diagnosed [25]. Regarding osteopontin alone as a marker, it was demonstrated in a different study that it performs better than AFP (AUROCs of 0.85 and 0.68, respectively), a benefit that remained for early-stage HCC; this is further discussed below [26].

An interesting study from Asia evaluated serum levels of aldo-keto reductase family 1 member B10 (AKR1B10) as a putative HCC biomarker. The biomarker detected early-stage HCC with a sensitivity of 61% and a specificity of 86% and performed better than AFP alone. However, the highest performance was achieved by the combination of both biomarkers (AUROC = 0.94). Such findings were similar in a validation cohort [27].

Finally, glypican-3 is a biomarker that has been evaluated in many studies. Glypican-3 is a transmembrane proteoglycan anchored to the cell membrane, highly expressed by some HCC tumors and can be detected in serum. Two recent meta-analyses have been published in which the value of serum glypican-3 levels in the diagnosis of HCC was evaluated. In the first study, glypican-3 performed similar to AFP (AUROCs of 0.78 and 0.79, respectively), while their combination had good accuracy (AUROC = 0.94) [28] in discriminating HCC from liver cirrhosis. The second meta-analysis evaluated the performance of glypican-3, Golgi protein 73 and AFP levels. These three biomarkers combined had an AUROC of 0.95 and performed better than any biomarker alone or any pair of biomarkers [29].

## 3. MicroRNA Biomarkers

Micro RNAs (miRNAs) are small non-coding RNA molecules of approximately 22–24 nucleotides in length that regulate gene expression and are critically involved in the processes of liver development during embryogenesis, liver homeostasis and liver pathophysiology [30]. miRNAs can be secreted into the extracellular space and are found circulating in various body fluids as either part of extracellular vesicles or exosomes (exo) or associated with circulating proteins [31]. Dysregulated expression of miRNAs has been demonstrated in various tumors, including the most common, such as lung, prostate, colon, breast and also liver cancers, and has been shown to affect the regulation of the activity of oncogenes and tumor suppressor genes, thereby directly influencing carcinogenesis [32]. As a consequence of their dysregulated expression, circulating miRNAs have been studied as potential biomarkers for cancer, including for HCC, detected using non-invasive techniques in serum or plasma [33]. miRNAs can be measured by molecular biology methods, like quantitative polymerase chain reaction (PCR), microarray or RNAseq analysis. Moreover, because miRNAs are small molecules, have a high sequence homology among family members and low abundance, new methods, such as those based on nanomaterials, are being developed for highly sensitive detection of miRNAs [34].

In a study from Korea [35], the expression levels of circulating miRNA were determined in serum of patients with HCC and in controls individuals with chronic HBV or liver cirrhosis. In this study, four exosome-derived miRNAs were found to be of interest (exo-miR-10b-5p, exo-miR-18a-5p, exo-miR-215-5p, and exo-miR-940) when examining serum samples from 90 patients with HCC and 60 controls with chronic liver disease. In particular, exo-miR-10b-5p appeared as a promising biomarker for early-stage HCC with an AUROC of 0.93, a sensitivity of 90.7% and specificity of 75.0% [35]. Another set of miRNAs, exo-miR-25-3p, exo-miR-1269a, exo-miR-4661-5p, and exo-miR-4746-5p with increased expression in HCC were found by selecting driver oncogenic miRNAs using analysis of miRNAs expression profiles from three different RNA sequencing datasets of human HCC [36]. In particular, serum exo-miR-4661-5p could detect HCC at all stages with AUROC of 0.92; even at early-stage HCC, the AUROC remained at 0.92. Furthermore, a panel composed of both exo-miR-4661-5p and exo-miR-4746-5p was able to detect early-stage HCC with an AUROC of 0.95, a sensitivity of 81.8% and a specificity of 91.7%. A retrospective study from China [37] identified a miRNA classifier containing seven circulating miRNAs (mIR-29a, mIR-29c, mIR-133a, mIR-143, mIR-145, mIR-192 and mIR-505) in serum that could detect HBV-induced HCC. This classifier showed higher accuracy than AFP (when using the 20 ng/mL cutoff) to distinguish individuals with HCC from individuals with chronic HBV or liver cirrhosis [37]. Interestingly, this study also established the ability of the miRNA classifier to predict preclinical HCC before diagnosis in a surveillance program with HBV. The miRNA panel detected 8 cases of HCC at 12 months before diagnosis (8 out of 27), whereas AFP could detect only 2 cases. A recent study evaluated the use of circulating miRNAs to identify HCC by analyzing serum samples from 353 HCC patients, 46 chronic hepatitis patients, and 93 patients with liver cirrhosis [38]. This study found that a combination of 8 miRNAs, miR-320b, miR-663a, miR-4448, miR-4651, miR-4749–5p, miR-6724–5p, miR-6877–5p, and miR-6885–5p could discriminate HCC from at-risk control samples (chronic hepatitis and/or cirrhosis) with a diagnostic value AUC of 0.99, a sensitivity of 97.7% and specificity of 94.7%. This model is proposed to detect 98% of stage I HCC cases [38]. The diagnostic value of this miRNA panel is superior to the diagnostic values achieved by either serum AFP, the GALAD score and the GALADUS score [20,21,22].

Since the various studies that examined the value of miRNA as an HCC biomarker examined either cell-free or exosomal derived miRNAs, a study involving 72 patients with HCC, 72 cirrhotic controls and 72 patients with HBV compared the diagnostic value and showed that a microRNA panel (miRNA-26a, miRNA-29c, and miRNA-21) in exosomes provided better diagnostic value for patients with HCC than circulating cell-free miRNAs among different groups [39]. Along the same line, it has been reported that the number of detected miRNA in plasma outnumbers the number detected in paired serum samples and that the expression levels in plasma were found to be higher than in serum, indicating that standardization of the biological material is crucial [40]. These observations may also, at least partially, explain that no research group has found the same discriminating set of miRNAs, with the exception of known dysregulated miRNAs in liver disease like miRNA-122 [41,42] or miRNA-21 [43,44] as candidates for early-stage HCC detection. Also, Table 1, which summarizes a series of microRNAs that are not further discussed in the review, clearly shows that most studies that evaluate circulating miRNAs as potential biomarkers for HCC detection were conducted using Asian cohorts, which are generally characterized by a high-frequency of HBV-induced HCC, and no or small numbers of HCC induced by HCV, alcohol or NAFLD. In addition, these studies were conducted involving small groups of patients and have yet to be validated within large cohort of patients [35,36,43,44,45,46,47].

Overall, circulating miRNAs appear to be promising biomarkers to detect early-stage HCC. To move this field further, longitudinal studies should be carried out, including larger cohorts to validate the diagnostic performance obtained by different sets of miRNAs in cross-sectional studies across different standardized detection platforms and disease etiologies.

## 4. Immune Biomarkers

Immunosurveillance, the recognition of tumor cells by leukocytes, has been well-described for many tumors and has changed the way to interpret the role of circulating immune markers in the setting of oncogenesis [66]. Hence, cytokines and chemokines induced upon recognizing cancerous lesions can be detected in serum or plasma of individuals at risk. This fact is highlighted in the formation of HCC, as the tumor usually arises in the setting of chronic hepatitis where a hyper-immune environment due to the continuous presence of an inflammatory response in the liver could lead to further alterations in measurable immune analytes during the transition from a liver nodule to HCC [67,68]. Our group recently identified a series of immune markers in serum of patients with hepatitis C that were associated with the future development of HCC, even when cancer occurred up to two years later [67]. These markers include, among others, soluble highly immunoreactive proteins, such as MIG, interleukin (IL)-22 and IL-3, as well as vascular endothelial growth factor (VEGF) and tumor necrosis factor-related apoptosis-inducing ligand (TRAIL), which are related to the vascular formation and apoptosis modulation. The c-statistic for the correct prediction of HCC was >0.90 in four of these markers (MIG, IL-22, TRAIL, APRIL), which is defined as extraordinary and >0.80 in the rest. However, this study was performed in a small number of samples (13 subjects per group), and all samples were from HCV-infected individuals. It is likely that in other chronic infections, such as HBV, these immune markers would play a role in early HCC detection as well.

Tumor growth factor β (TGF-β), a polyfunctional growth factor that has been shown to interact literally on all cell types by modulating cell proliferation, cell differentiation and even cell survival, also has potential as a biomarker for HCC. Mutations and differential expression of TGF-β have been found to be altered in a variety of tumor types. Previous studies have shown that serum levels of TGF-β are associated with HCC development, mainly in HCV-infected individuals [69]. However, other studies have addressed TGF-β role as a biomarker in HCC in combination with the expression of other proteins or mRNA and not as a stand-alone biomarker [70]. Moreover, several of these studies have been performed in single-country populations without confirmation in other settings.

Osteopontin (OPN), a versatile cytokine, which mediates a wide array of biological functions in the immune and vascular system, has been reviewed before as a marker for a variety of tumors [71]. Several studies have shown increased serum and plasma levels of OPN in individuals with HCC compared to those with liver cirrhosis and/or chronic liver disease controls [26,72,73]. The majority of these studies were carried out in Asian cohorts, with a large multicenter study conducted using West-African and European cohorts replicating these findings [74]. In most studies, OPN has shown an AUROC of no less than 0.75 for HCC prediction. In contrast, the diagnostic efficacy of OPN in detecting early-stage HCC vs. non-HCC patients varied considerably depending on the study. Ge et al. [75] and Vongsuvanh et al. [76] reported an AUROC of 0.57 and 0.78, respectively, whereas Shang et al. reported an AUC of 0.73 [73]. Interestingly, a prospective evaluation in an Asian cohort of 115 patients with chronic liver disease at risk of HCC showed increased plasma OPN levels 24 months prior to diagnosis in 21 subjects who developed HCC [74].

Recently, serum pentraxin 3 also has been suggested as a candidate biomarker of HBV-induced HCC in a study from China [77]. Pentraxin 3 is a protein produced by multiple cell types, such as macrophages, monocytes, fibroblasts and endothelial cells in response to inflammatory signals (such as bacterial components or cytokines, such as TNF or IL-1), and as such, pentraxins behave as acute-phase proteins. Pentraxin 3 may also be involved in cancer development, although the underlying mechanisms are not well understood. Elevated pentraxin 3 levels have been reported in patients with acute liver injury, NASH and HCV, among others. Evaluating the serum pentraxin 3 levels in 107 patients with HCC in comparison to 159 chronic HBV and 99 cirrhotic patients demonstrated that pentraxin 3 was highly discriminative of AFP-negative and early-stage HCC, and the diagnostic performance of pentraxin 3 was superior to AFP. In fact, the AUC for pentraxin to discriminate early HCC from cirrhosis was 0.90, while it was 0.68 for AFP, clearly suggesting the potential of pentraxin 3 as a biomarker for early HCC.

Chemokines play an important role as mediators of immune responses since they are instrumental in the recruitment and activation of leukocytes at the inflamed or injured location. The chemokines C–C motif ligand 4 (CCL4) and CCL5 bind to the same receptor, C–C receptor 5, which is expressed in effector and memory T cells, making this interaction critical in controlling chronic viral infections [78]. Only one study to date has evaluated serum levels of various chemokines in the context of HCC detection, and multivariate regression analysis found that serum CCL4 and CCL5 levels were higher in cirrhotics with HCC than cirrhotics without HCC (*n* = 78), making them interesting candidate diagnostic markers for HCC. The performance of CCL4 and CCL5 was comparable for HCC detection, with an AUROC for CCL5 of 0.72 and relatively high sensitivity of 71% and specificity of 68% [76].

VEGF, an angiogenic factor for vascular endothelial cells, is produced by many cell types, including tumor cells [79]. A Japanese study [80] involving HCV-infected individuals with HCC demonstrated that the AUROC for detection of HCC of VEGF was superior to that of AFP (AUROC of 0.98 versus 0.71, respectively) [80]. However, a study performed in Egypt, also in HCV-infected individuals (all genotype 4), did not detect serum VEGF differences among HCV patients who developed HCC and control HCV patients [81]. The recent longitudinal study from our group, mentioned above, identified serum VEGF as one out 12 immune mediators to be increased in HCV-induced HCC [67]. Despite a promising status, VEGF requires further investigation as a stand-alone marker for early or late HCC detection.

Multiple studies have shown a role for IL-6 in inflammation leading to liver cancer, and even gender disparities in HCC have been explained by the interrelation between estrogen and IL-6 [82]. Serum IL-6 has been shown to be increased in HCC patients compared to patients with chronic liver disease [83]. However, most studies have evaluated the performance of IL-6 in the setting of advanced HCC [84], but not during the early stages. Of note, IL-6 pretreatment levels did not associate with macrovascular invasion or extrahepatic spread, but some studies show a potential for this cytokine as a predictor to response to systemic therapy [84,85]. Moreover, cellular models have described decreased resistance to sorafenib by inhibiting IL-6-related pathways [85].

Growth differentiation factor 15 (GDF15), a member of the TGF-β superfamily, has been shown to be elevated in HCC compared to controls in HBV- and HCV-positive Chinese cohorts [86]. Although this study initially raised promise about the perspectives of GDF15, a later study found increased serum levels of the immune analyte in HBV-related HCC and HCV-related HCC compared to either chronic viral infection, but no statistical difference when compared to cirrhotic patients [87]. Further studies performed in prospective cohorts are needed to assess the role of GDF15 in HCC as well as in non-viral hepatitis-related HCC.

## 5. Conclusions

One of the main factors related to the high mortality in HCC is the frequency of late diagnosis of this tumor. Despite attempts to implement surveillance strategies, adherence to surveillance programs has been reported to be low across the globe [88]. Peripheral biomarkers that are easily measured in serum or plasma are of critical need in the field of HCC. However, the tumor’s genetic diversity has blunted efforts to discover reliable and specific proteins that could aid in the early detection of HCC. Moreover, the conundrum of liver diseases underlying the development of liver cirrhosis and hence HCC, such as viral hepatitis, create an immune imbalance that further obscures the ability to identify immune markers or peripherally expressed proteins that could assist in early detection. However, recent advances in techniques allowing the detection of multiple immune analytes, as well as the progress in quantifying microRNAs specific to the liver, have contributed to a change in balance that favors the proper implementation of biomarkers, which easily and effectively predict or diagnose early HCC. Moreover, new technologies that allow for exosome (endovesicles) assessment in relation to HCC detection will bring this field to new territories. It should be expected that in the next 5–10 years, the hepato-oncology community will have a broader spectrum of tools to predict HCC with a simple serum assessment.

## Figures and Tables

**Table 1:** Summary of published literature on miRNA evaluation as a biomarker for hepatocellular carcinoma (HCC).

miRNA	Etiology	Country	Source	Study Subjects	Reference
miR-21, miR-106b, miR-125b, miR-182, miR-224	Multiple	China	Serum	66 HCC patients and 82 healthy controls	[48]
miR-21, miR-26a miR-101	Multiple	China	Serum	52 HCC patients, 42 chronic hepatitis patients and 43 healthy controls	[49]
miR-88	HBV	China	Serum	80 HCC patients, 45 liver cirrhosis patients, 45 chronic HBV patients and 43 healthy controls	[50]
miR-375, miR-10a, miR-122, miR-423	Multiple	China	Serum	149 HCC patients and 149 controls	[51]
miR-122, miR-125b, miR-145, miR-192, miR-194, miR-29a, miR-17-5p, miR-106a	Multiple	China	Serum exosomes	80 HCC patients and 30 healthy controls	[47]
miR-122, miR-224	HCV	Egypt	Plasma	40 HCC patients related to HCV, 40 chronic HCV patients and 20 healthy volunteers	[52]
miR-21, miR-122	Multiple	India	Serum	50 HCC patients, 25 chronic hepatitis patients, 25 liver cirrhosis patients and 10 healthy individuals	[53]
miR-122, miR-148a, miR-1246 miR-486, miR-584	Multiple	China	Serum exosomes	68 HCC patients, 53 liver cirrhosis patients, 50 chronic hepatitis patients, 64 controls	[54]
miR-19a, miR-296, miR-195, miR-192, miR-34a, miR-146a	HCV	Egypt	Serum	112 HCC patients related to HCV, 125 chronic HCV patients and 42 healthy controls	[55]
miR-375 miR-199a-3p	Multiple	China	Serum	78 HCC patients and 156 healthy controls	[56]
miR214-5p, miR125b, miR-1269 miR-375	HCV	Egypt	Serum	224 HCC patients related to HCV, 250 chronic HCV patients and 84 healthy controls	[57]
miR-4463	HBV	China	Serum	45 HCC patients and 45 controls	[58]
mIR-106b	Not mentioned	China	Serum	335 HCC patients	[59]
miR-10b, miR-106b, miR-181a	Multiple	China	Serum	27 HCC patients, 31 chronic liver disease patients and 50 healthy controls	[60]
miR-23a	Not mentioned	Egypt	Serum	57 HCC patients, 57 liver cirrhosis patients and 57 healthy controls	[61]
miR-10b-5p miR-215–5p	HBV	Korea	Serum exosomes	90 HCC patients, 60 chronic liver disease patients and 28 healthy controls	[35]
miR-301	HCV	Egypt	Plasma	42 HCC patients related to HCV, 48 chronic HCV patients (all with liver cirrhosis) and 40 healthy controls	[62]
miR-1246, miR-101–3p miR-106b-3p	Multiple	Italy	Plasma	cohort 1:7 HCC patients, 10 liver cirrhosis patients and 7 controls; cohort 2:9 HCC patients and 6 liver cirrhosis patients cohort 3:22 HCC patients and 11 healthy controls	[45]
miR-224	HBV	China	Serum	182 HCC patients	[63]
miR-150, miR-182	HCV	Egypt	Serum	40 HCC patients, 40 chronic HCV patients (20 cirrhotic and 20 non-cirrhotic) and 40 healthy controls.	[64]
miR-122, miR-221, miR-222 miR-224	HBV	China	Serum exosomes	20 HCC patients, 20 chronic HBV patients, and 20 liver cirrhosis patients	[65]

## Data Availability

The data presented in this study are available on request from the corresponding author.
